# Practical Advancement of Multipollutant Scientific and Risk Assessment Approaches for Ambient Air Pollution

**DOI:** 10.1289/ehp.1204939

**Published:** 2012-05-29

**Authors:** Douglas O. Johns, Lindsay Wichers Stanek, Katherine Walker, Souad Benromdhane, Bryan Hubbell, Mary Ross, Robert B. Devlin, Daniel L. Costa, Daniel S. Greenbaum

**Affiliations:** 1 National Center for Environmental Assessment, Office of Research and Development, U.S. Environmental Protection Agency, Research Triangle Park, North Carolina, USA; 2 Health Effects Institute, Boston, Massachusetts, USA; 3 Office of Air Quality Planning and Standards; 4 National Health and Environmental Effects Research Laboratory, Office of Research and Development, and; 5 Office of Research and Development, U.S. Environmental Protection Agency, Research Triangle Park, North Carolina, USA

**Keywords:** air pollution, exposure, human health, multipollutant, risk assessment

## Abstract

Objectives: The U.S. Environmental Protection Agency is working toward gaining a better understanding of the human health impacts of exposure to complex air pollutant mixtures and the key features that drive the toxicity of these mixtures, which can then be used for future scientific and risk assessments.

Data sources: A public workshop was held in Chapel Hill, North Carolina, 22–24 February 2011, to discuss scientific issues and data gaps related to adopting multipollutant science and risk assessment approaches, with a particular focus on the criteria air pollutants. Expert panelists in the fields of epidemiology, toxicology, and atmospheric and exposure sciences led open discussions to encourage workshop participants to think broadly about available and emerging scientific evidence related to multipollutant approaches to evaluating the health effects of air pollution.

Synthesis: Although there is clearly a need for novel research and analytical approaches to better characterize the health effects of multipollutant exposures, much progress can be made by using existing scientific information and statistical methods to evaluate the effects of single pollutants in a multipollutant context. This work will have a direct impact on the development of a multipollutant science assessment and a conceptual framework for conducting multipollutant risk assessments.

Conclusions: Transitioning to a multipollutant paradigm can be aided through the adoption of a framework for multipollutant science and risk assessment that encompasses well-studied and ubiquitous air pollutants. Successfully advancing methods for conducting these assessments will require collaborative and parallel efforts between the scientific and environmental regulatory and policy communities.

In a recent published commentary (Mauderly et al. 2010), a group of scientists representing academia, government, and industry groups posed the question, “Is the air pollution health research community prepared to support a multipollutant air quality management framework?” In agreement with several other contemporary reviews, editorials, and opinion papers on the subject (e.g., Dominici et al. 2010; Greenbaum and Shaikh 2010; Vedal and Kaufman 2011), the authors concluded that, although significant data gaps limit our current understanding of health effects resulting from exposure to air pollutant mixtures, much can be gained in the near future through an increased emphasis on multipollutant issues across the spectrum from basic scientific research through implementation of air quality control strategies. Although single-pollutant approaches to air pollution research, health assessments, and setting standards for ambient air quality have been successful in reducing air pollution over the past few decades, there is a clear need for parallel efforts within both the scientific and the regulatory and policy communities to advance methods for evaluating and managing the effects of air pollution using a multipollutant approach.

This review derives from a multipollutant science and risk analysis public workshop that was held on 22–24 February 2011 in Chapel Hill, North Carolina, with the purpose of providing a brief overview of the state of the science and identifying data gaps related to addressing the health consequences of air pollution in a multipollutant context, including realistic steps and targets to advancing scientific and policy decisions. Co-organized and sponsored by the U.S. Environmental Protection Agency (EPA) and the Health Effects Institute (HEI), this workshop was designed to facilitate open discussions among expert scientists; these discussions are now playing a key role in multipollutant research planning within the U.S. EPA’s Office of Research and Development and are also helping to guide the development of the U.S. EPA’s framework for conducting multipollutant science and risk assessments.

Evaluating the health impacts of multipollutant exposures has been identified as a priority research area in the U.S. EPA’s integrated, cross-disciplinary research planning (U.S. EPA 2012), including the establishment of four university-based Clean Air Research Centers (CLARCs) to study exposures to air pollution mixtures and their associated health effects. As additional evidence of its commitment to this new thinking, scientists within the U.S. EPA’s National Center for Environmental Assessment (NCEA), which is responsible for evaluating and synthesizing the scientific information related to the effects of exposure to criteria air pollutants as a part of the National Ambient Air Quality Standards (NAAQS) review process, are currently developing plans for conducting a formal multipollutant science assessment (MSA) of the health effects of exposure to air pollutant mixtures. As an initial step in the development of this proposed human health MSA, the U.S. EPA is preparing a framework describing the purpose and scope of the MSA, along with plans for conducting multipollutant analyses using existing data and information that will provide scientific support to the development of the MSA. The MSA is intended to serve as a companion document to single-pollutant Integrated Science Assessments (ISAs) of the criteria air pollutants (i.e., particulate matter, ozone, nitrogen oxides, sulfur oxides, lead, and carbon monoxide), and allow for a more effective evaluation of both the health effects of air pollutant mixtures, as well as the effects of single pollutants in a multipollutant context. This approach is consistent with the recommendations from the 2004 National Research Council (NRC) Report, *Air Quality Management in the United States* (NRC 2004):

Although the committee does not believe that the science has evolved to a sufficient extent to permit development of multipollutant NAAQS [National Ambient Air Quality Standards], it would be scientifically prudent to begin to review and develop NAAQS for related pollutants in parallel and simultaneously.

It is anticipated that the development of the MSA may eventually inform multipollutant risk assessment; however, conducting such a risk assessment has been one of the main challenges in multipollutant science and policy (Brook et al. 2009). Technologies for exposure assessment, knowledge of exposure–response relationships, as well as the ability to communicate risk to different stakeholders are key challenges for multipollutant risk assessment. A conceptual framework that will address risk assessment in a multipollutant context is currently under consideration by staff within the U.S. EPA’s Office of Air and Radiation (OAR). However, the introduction of this conceptual framework is not an indicator that the use of multipollutant risk assessment is imminent. Instead, this conceptual scheme would probe the current technology, modeling tools, and the data available to help design building blocks for the implementation of a multipollutant risk assessment. Figure 1 outlines the essential elements in air pollution risk assessment, beginning with emission sources, pollutant transport and transformation, and control. In addition to source-specific emissions, global emissions (including transboundary transport and transformation) impact regional air quality and thus contribute to overall risk. The middle section of this figure identifies traditional pillars of risk assessment that are heavily reliant on research and data collection through monitoring, modeling, and experimental evaluation of health impacts in support of assessing risk. At the base of Figure 1, the different approaches that are used to characterize adverse biological responses are provided: concentration–response curves that are obtained through observational data such as epidemiologic studies; exposure–response functions based on modeling or personal exposure studies; and dose–response curves derived from controlled human exposure studies or observational studies using biomarkers of exposure. The weight of evidence for interpreting risk results requires recognition of these different approaches that depend on the properties of the pollutant of interest and the data available.

**Figure 1:**
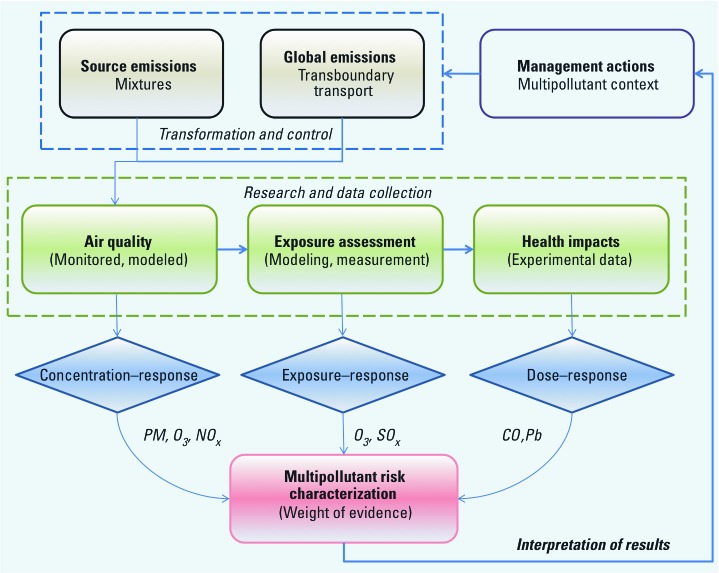
Conceptual scheme for multipollutant risk assessment. Abbreviations: CO, carbon monoxide; NO_x_, nitrogen oxides; Pb, lead; PM, particulate matter; O_3_, ozone; SO_x_, sulfur oxides.

The complexities involved in the development of multipollutant science and risk assessments are considerable, and will require scientists from various disciplines and organizations to work more collaboratively going forward. For the purpose of this review, “multipollutant” is generally defined as the criteria air pollutants along with some priority air toxics. Although this approach does not include all ambient air pollutants to which populations are exposed, it does represent a relatively manageable set of pollutants. Further, the health impacts of exposures to the criteria air pollutants have been extensively studied, thus making them an ideal starting place when considering a multipollutant approach to evaluating the health effects of exposure to air pollution.

## Evaluating the Human Health Effects of Multipollutant Exposures

*Human exposures.* Characterizing exposure to multipollutant mixtures requires an advanced understanding of the sources of air pollutants, the chemical transformations and interactions between multiple pollutants, and information on the correlations in space and time between their individual concentrations. The most relevant consideration for risk assessment is how different mixtures contribute to the overall exposures of populations, which are governed by the time spent in contact with each mixture and its constituent component pollutants. Assessing multipollutant exposure will be aided by the ability to distinguish which mixtures or parts of mixtures are most closely associated with particular health outcomes, rather than assessing exposure to all possible mixtures. Once the most relevant mixtures are identified, it is also possible to study the origins of those mixtures including transport and transformation of emissions from those sources to exposure environments where populations receive significant exposures. Although the complexity of the mixture can be considerable, recognizing and appreciating this complexity may, in the end, allow assessments to be conducted with information going beyond a finite set of what seem to be the so-called “most relevant” mixtures.

Several innovative, albeit ambitious, ideas have been proposed to better characterize exposures to air pollutant mixtures. Approaches involving the use of existing data may center around grouping air pollutants based on sources, microenvironments, or chemical and physical properties (e.g., Suh et al. 2011). In developing these groupings, it is essential that atmospheric and exposure scientists work collaboratively with health scientists to consider approaches informed by pollutant-specific toxicological pathways. Source apportionment techniques may be useful in characterizing the contributions to personal exposure from particular emission sources. However, specific source categories can be difficult to identify given multiple sources of pollutants, their differing spatial distribution relative to monitors, and the formation of secondary air pollutants (Brinkman et al. 2009). In addition, the composition and concentrations of some source emissions change over time due to technological and regulatory factors (e.g., changes in diesel engines), as well as to economic influences (e.g., changes in the prices of fuels). Although many sources and exposure environments may be important, existing and supplementary monitoring evidence suggests that an initial subset of key source categories, including ports, roadways, industrial centers, and biomass burning can form the basis for studies in the nearer term, while others may be identified in future source apportionment studies.

It is clear that neither modeling nor additional monitoring alone is likely to solve the problem of characterizing multipollutant exposures. For example, while grid modeling techniques have the potential to address uncertainties related to spatial variability of air pollutant mixtures, in many cases these models do not provide adequate temporal resolution, require detailed source and meteorological data inputs, and are difficult to evaluate (Marmur et al. 2006). Similarly, efforts to reduce complexity by use of a measured chemical marker species to represent a given source or microenvironment have clear advantages, but are likely overly simplistic due to differences in monitor location and frequency of data collection that vary by pollutant (termed spatial and temporal misalignment, respectively) (Gryparis et al. 2009). However, one potentially promising approach to reduce uncertainties associated with spatial and temporal misalignment is the integration of monitoring data, as well as satellite imagery, with modeling techniques that provide interpolation of atmospheric concentrations, potentially resulting in a more complete characterization of spatial and temporal variations of individual pollutant and combined pollutant exposures (Liu et al. 2005; Villeneuve et al. 2011).

With respect to monitoring data, trade-offs must inevitably be made between quantity and quality of the data. For example, the development of inexpensive personal air quality sensors has considerable appeal as an approach to gathering personal exposure data, but equal concern exists about the analytical sensitivity and accuracy of these monitors. However, if sensors are deployed widely, patterns of pollutants and variations in time and space may be invaluable in model development despite shortcomings in accuracy. Recognizing the limitations of personal monitoring and uncertainties associated with the use of central-site monitors to represent personal exposure to ambient air pollutants, the implementation of an exposure supersite program may be warranted. At such a site, the spatial and temporal variability of the concentrations of multiple pollutants would be characterized in multiple microenvironments using similar sampling methods. Although each of these approaches has merit, adequately characterizing exposures to air pollutant mixtures to support multipollutant risk assessments may best be accomplished through integration of central site monitors, widely deployed sensors, satellite measurements, and refined atmospheric chemistry and exposure modeling that can draw across the relative strengths of each approach, to allow for focused validation and tool development.

*Health studies.* The development of a MSA for health effects will entail extensive integration of findings from observational, experimental, and exposure studies. For observational studies, moving toward a multipollutant focus will require drawing upon many established epidemiologic approaches. Single-pollutant models can be used to evaluate one pollutant as an indicator of a group of pollutants, and under certain conditions, traditional two-pollutant models can provide insight into combined effects through summing model coefficients or partial derivatives (Bateson et al. 2007). Epidemiologic studies that rely on source apportionment methods to characterize exposure are another way to gain insight into the relationship between health effects and a group of correlated pollutants.

Generally, there are three broad approaches to evaluating interactions or other types of effect modification in epidemiologic studies—traditional regression models, dimension reduction techniques, and Bayesian hierarchical methods (Billionnet et al. 2012). All have their merits, which include ease of use for the former two approaches and flexibility for the latter approach. Future approaches may borrow and build on newer techniques from the genomics and other “omics” communities, applied in a multipollutant context, including clustering methods and random forest approaches (Breiman 2001; Siroux et al. 2011).

Monitoring networks will need to provide enhanced capabilities to evaluate temporal and spatial scales. Spatial considerations include improved resolution of exposure and a better understanding of the representativeness of central-site monitors. Spatial misalignment of exposure data is an issue when evaluating multipollutant effects because the ability to estimate exposures in different locations for different pollutants varies. Observational studies already consider some temporal patterns of health outcomes; for example, daily concentrations (or < 24 hr) are necessary for assessing acute effects and long-term concentrations (including seasonal variation) are required for assessing more chronic effects. Moving forward, the development of statistical methods that allow for clearer separation of the impacts of temporal and spatial variation may enable the resolution of multipollutant effects (Bell et al. 2007; Peng and Bell 2010). These types of methods may also be valuable in understanding why changes are observed in the magnitude of risks associated with individual pollutants over time. We can hypothesize that these changes may be due to changing levels and mixes of pollutants, potentially due to new regulations on diesel engine and power plant emissions, but additional research is needed to test these hypotheses.

Experimental studies with animals or intentional environmental exposures involving humans will likely continue to provide evidence of biological plausibility for the impact of mixtures as they do for single pollutants. Because they are hypothesis driven, they can be used to directly identify individual causal agents and their contributions to an overall effect. Although information on effects occurring at ambient levels is most relevant when considering human health risks, differences in respiratory tract deposition and biological response between animals and humans may justify the use of exposure concentrations that exceed ambient concentrations in animal toxicology studies (Brown et al. 2005). Various experimental approaches are currently available to evaluate multipollutant effects. One such approach involves animal and human exposures to “real world” mixtures of air pollutants under controlled conditions or environments that simulate ambient conditions, such as in photochemical chambers (Lemos et al. 2011; Sexton et al. 2004) or a traffic tunnel (Kooter et al. 2006). One limitation of this type of study is its inability to precisely control or manipulate the exposure concentrations or pollutant mixture. The potential strength of this study design resides in the spatial and temporal contrasts for exposure and response. Another approach is laboratory-generated mixture studies where a few pre-selected individual pollutants are combined to create an exposure atmosphere, with the most informative studies being those that have an air control as well as exposures to individual pollutants (Mauderly and Samet 2009). Although the mixtures being studied do not reflect the aging and transformation that occur under ambient conditions, they do provide relevant information on the nature of the interactions among different chemicals.

A mode-of-action (MOA) framework describing a sequence of key events leading to an end point or clinical outcome represents a possible unifying theme when considering multiple pollutants and the common toxicity pathways through which they act (Ankley et al. 2010). Such a framework should make it possible to characterize and illustrate the interconnectedness of key event pathways and the upstream responses that produce those events. The focus of the framework should be on commonalities between air pollutants, such as robust toxicity pathways informed by well established measurements of phenotypic changes and biomarkers linking exposure and effect. This approach also has the capability of evaluating the nature of interactions between pollutants and identifying potentially susceptible populations.

Thus, two possible approaches to address pollutant interactions are currently being developed: the common occurrence approach where a related group of pollutants from a particular source or resulting atmospheric mixture are associated with a health end point, and the MOA approach that groups specific chemicals in the atmosphere based on their effects with the same health end point. Limitations in toxicological approaches and epidemiologic data and statistical methods will require cross-disciplinary hypothesis development, research planning, and execution of this research (Dominici et al. 2008).

## Synthesis and Discussion

The historical focus on single pollutants has brought us a long way—a substantial data base of exposure monitoring data; a large and growing body of evidence on different facets of the MOA for individual pollutants; a diverse toolbox of toxicological, clinical, and epidemiologic study designs; statistical and other data analysis methods; and air quality models that we can rely on in the short term. But it is also clear that we have a ways to go—whether it is toward the adaptation of existing data and tools or toward the development of new ones. In short, the transition to a deliberative multipollutant approach will require substantial research effort and time. The U.S. EPA’s current efforts to develop a MSA, along with plans for designing a framework for conducting multipollutant risk assessments, provide an important structure and context for assessing the strengths and limitations of the existing science related to the health effects of exposures to air pollutant mixtures. In addition, the current and planned U.S. EPA and U.S. EPA-funded multipollutant scientific research is clearly vital in making a successful transition from single to multipollutant approaches to air quality evaluation and management.

The U.S. EPA began using a multipollutant approach in a limited context in the recent evaluation of the secondary standards for nitrogen oxides and sulfur oxides. The development of a U.S. EPA policy assessment for nitrogen oxides and sulfur oxides resulted in consideration of a multipollutant NAAQS to protect against the combined effects of nitrogen and sulfur oxides on aquatic acidification (EPA 2011). OAR also has recently conducted a pilot study that demonstrated how multipollutant approaches to implementing primary (human health–based) air pollution standards could result in greater health benefits and more cost-effective implementation, which may also help to achieve greater reductions in air toxics when compared with single-pollutant approaches (Fann et al. 2011). The adoption of a multipollutant policy for the review of ambient air pollutants would potentially result in increased efficiencies, benefits, and cost savings throughout the process beginning with the initial evaluation of the scientific evidence. More important, increasing the emphasis on multipollutant approaches may allow for a better understanding of the types of air pollutant mixtures most likely to result in adverse health effects, which could, in turn, facilitate the identification of control strategies to minimize exposures to these mixtures.

**Figure 2:**
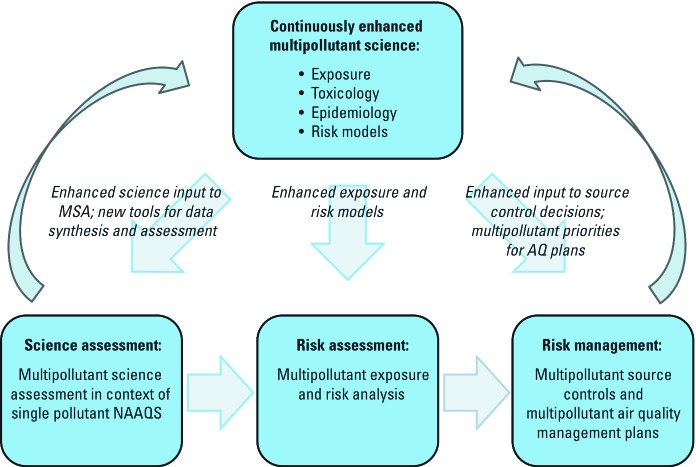
Schema showing continuous enhancement of multipollutant science.

As the science, risk assessment, and risk management communities advance toward adopting multipollutant approaches, it is important to set realistic targets for progress amidst a very large number of possible directions for improvement. We should aim to take concrete steps in that direction in at least four major areas (Figure 2):

Exposure—seeking to increase the scientific base of health studies in which multiple pollutants are measured simultaneously in ambient air
Toxicology—seeking enhanced measurement and analysis techniques for source and ambient mixture (e.g., concentrated ambient particles) exposure, and better understanding of common MOAs (key events) as a useful way to group and assess pollutants
Epidemiology and statistics—seeking enhanced techniques, especially statistical analysis techniques, that enable the characterization of associations between multiple pollutants (i.e., more than two at a time), sources, and health outcomes
Modeling and risk assessment—seeking the development and testing of multipollutant approaches to estimating population exposure and risk.

Enhanced science in each of these areas can inform the three major components of air quality management—science assessment, risk assessment, and risk management (as in Figure 2). Additional scientific questions may arise leading to new scientific developments, further supporting progress toward understanding effects of joint exposures to multiple pollutants (e.g., the criteria pollutants and major air toxics) and, if the data permit, identifying individual pollutants and sources within the mixture that may be disproportionately responsible for adverse effects on human health.
